# AMP-activated protein kinase preserves endothelial tight junctions in the coronary microcirculation during sepsis

**DOI:** 10.1186/cc11717

**Published:** 2012-11-14

**Authors:** D Castanares-Zapatero, C Bouleti, T Mathivet, B Gerber, C Oury, L Bertrand, JL Vanoverschelde, PF Laterre, S Horman, C Beauloye

**Affiliations:** 1 Université catholique de Louvain, Institut de recherche expérimentale et clinique (pôle cardiovasculaire), Brussels, Belgium; 2 Collège de France, Centre Interdisciplinaire de Recherche en Biologie INSERM 1050, Paris, France; 3 Université de Liège, Groupe Interdisciplinaire de Génoprotéomique Appliquée, Liège, Belgium; 4 Université catholique de Louvain, Cliniques universitaires Saint Luc Intensive Care Unit, Brussels, Belgium

## Background

A compromising endothelial cell (EC) monolayer affects vascular permeability and leads to fluid extravasation. Tight junctions and more particularly Zonula occludens 1 (ZO-1) play a major role in maintaining vascular barrier integrity and are regulated by cytoskeletal proteins. During sepsis, endothelial barrier disruption occurs in most organs and contributes to organ dysfunction. We and others have demonstrated that the α_1 _isoform of AMP-activated protein kinase (α_1_AMPK) controls cytoskeleton organisation in various cell types including endothelial cells. Therefore, we hypothesised that α_1_AMPK preserves tight junction organisation and vascular permeability during sepsis in the coronary microcirculation.

## Methods

*In vitro*, tight junction organisation (ZO-1 staining), cytoskeleton organisation (phalloïdin staining) and vascular permeability were measured in endothelial cells in culture (HCAECs). Endothelial cells were pretreated with 1 mM AICA riboside (AICAr) before LPS challenge (50 μg/ml O55:B5). *In vivo*, wild-type (α_1_AMPK^+/+^) mice were treated with LPS (O55:B5,10 mg/kg) and compared with α_1_AMPK knockout animals (α_1_AMPK^-/-^). ZO-1 localisation was determined on frozen heart sections. Vascular permeability was evaluated using Evans Blue dye leakage. In addition, myocardial wall oedema was assessed by magnetic resonance imaging (MRI).

## Results

*In vitro*, LPS-challenged cells displayed a significant disruption of the ZO-1 linear configuration after 24 hours and exhibited a decrease in peripheral actin filaments. Gap areas appeared in the cellular monolayer exposed to LPS, unlike untreated cells for which the monolayer remained unaltered. Consequently, LPS treatment gradually increased endothelial cells monolayer permeability in a time-dependent manner. AMPK activation by AICAr preserved the linear pattern of ZO-1 and prevented gap areas in the monolayer. More interestingly, AMPK activation reduced LPS-induced hyperpermeability. *In vivo*, according to the *in vitro *experiments, a dramatic decrease in ZO-1 staining was observed in α_1_AMPK^-/- ^hearts compared with α_1_AMPK^+/+^, after LPS challenge. This resulted in an increased Evans Blue extravasation and, more importantly, in an exaggerated myocardial wall oedema observed by MRI. Finally, treating C57Bl6 mice with AICAr reduced cardiac vascular hyperpermeability in our model of sepsis.

## Conclusion

The AMPK signalling pathway protects the endothelial barrier during sepsis by preserving tight junction organisation. Since AMPK counteracts the molecular events involved in the LPS-induced barrier disruption in coronary microcirculation, it could consequently represent a new therapeutic molecule during sepsis.

